# Human melanoma cells inhibit the earliest differentiation steps of human Langerhans cell precursors but failed to affect the functional maturation of epidermal Langerhans cells

**DOI:** 10.1054/bjoc.2001.2183

**Published:** 2001-12

**Authors:** O Berthier-Vergnes, M Gaucherand, J Péguet-Navarro, J Plouet, J-F Pageaux, D Schmitt, M-J Staquet

**Affiliations:** INSERM U 346, Hôpital Edouard Herriot, Lyon, F-69437, France; UPR CNRS 9062 IPBS, F-31077, Toulouse, France; INSERM U352-INSA, F-69621, Villeurbanne, France

**Keywords:** human melanoma, Langerhans cell, differentiation, maturation

## Abstract

Tumour-derived factors suppress differentiation and function of in vitro generated DC. Here, we investigate the effect of two melanoma clones differing in their invasive and metastatic properties on the generation and/or functional maturation of human epidermal LC. LC were generated from CD34^+^ cord blood progenitors under GM-CSF/TNF-α/TGF-β1. CD34^+^ cells were co-cultured with or without melanoma cells using Transwell dishes. After 11 days of co-culture, CD34^+^-derived cells display a non-adherent undifferentiated morphology, a high level of monocytic CD14 marker, a down-regulated expression of LC markers (CD1a, E-cadherin) and DC markers (CD40, CD80, CD54, CD58, CD83, CD86, HLA-DR, HLA-class I). These cells were less potent than control LC in inducing allogeneic T cell proliferation. The generation of the CD14^+^ population was correlated with a decrease in the CD1a^+^ population, without any statistical differences between the two clones. Melanoma cells diverted the differentiation of CD34^+^ cells towards a dominant CD14^+^ population only if the progenitors were in an early growth phase. IL-10, TGF-β1 and VEGF were not responsible for these effects, as assessed by using blocking antibodies. By contrast, co-culture of fresh epidermal LC with melanoma cells did not affect their phenotype and function. Our data demonstrate that melanoma cells inhibit the earliest steps of LC differentiation, but failed to affect the functional maturation of epidermal LC. This suggests that melanoma cells participate in their own escape from immunosurveillance by preventing LC generation in the local cutaneous microenvironment. © 2001 Cancer Research Campaign http://www.bjcancer.com


British Journal of Cancer (2001) 85(12), 1944–1951
© 2001 Cancer Research Campaign
doi: 10.1054/ bjoc.2001.2183, available online at http://www.idealibrary.com on IDEAL

http://www.bjcancer.com

### Human melanoma cells inhibit the earliest differentiation steps of human Langerhans cell precursors but failed to affect the functional maturation of epidermal Langerhans cells

**O Berthier-Vergnes[1], M Gaucherand[1], J Péguet-Navarro[1], J Plouet[2], J-F Pageaux[3], D Schmitt[1] and M-J Staquet[1]**

[1]INSERM U 346, Hôpital Edouard Herriot, F-69437 Lyon, France; [2]UPR CNRS 9062 IPBS, F-31077 Toulouse, France; [3]INSERM U352-INSA, F-69621 Villeurbanne, France

**Summary** Tumour-derived factors suppress differentiation and function of in vitro generated DC. Here, we investigate the effect of two melanoma clones differing in their invasive and metastatic properties on the generation and/or functional maturation of human epidermal LC. LC were generated from CD34[+] cord blood progenitors under GM-CSF/TNF-α/TGF-β1. CD34[+] cells were co-cultured with or without melanoma cells using Transwell dishes. After 11 days of co-culture, CD34[+]-derived cells display a non-adherent undifferentiated morphology, a high level of monocytic CD14 marker, a down-regulated expression of LC markers (CD1a, E-cadherin) and DC markers (CD40, CD80, CD54, CD58, CD83, CD86, HLA-DR, HLA-class I). These cells were less potent than control LC in inducing allogeneic T cell proliferation. The generation of the CD14[+] population was correlated with a decrease in the CD1a[+] population, without any statistical differences between the two clones. Melanoma cells diverted the differentiation of CD34[+] cells towards a dominant CD14[+] population only if the progenitors were in an early growth phase. IL-10, TGF-β1 and VEGF were not responsible for these effects, as assessed by using blocking antibodies. By contrast, co-culture of fresh epidermal LC with melanoma cells did not affect their phenotype and function. Our data demonstrate that melanoma cells inhibit the earliest steps of LC differentiation, but failed to affect the functional maturation of epidermal LC. This suggests that melanoma cells participate in their own escape from immunosurveillance by preventing LC generation in the local cutaneous microenvironment. © 2001 Cancer Research Campaign http://www.bjcancer.com

**Keywords**: human melanoma; Langerhans cell; differentiation; maturation

Dendritic cells (DC) are bone marrow-derived professional antigen-presenting cells (APC) that are required for the initiation of immune responses. They exhibit a broad heterogeneity in their anatomical location, migration pathways, phenotype and functional abilities. Langerhans cells (LC) are the DC of the epidermis characterized by the expression of CD1a, E-cadherin and Birbeck granules that distinguish them from other DC members (Hart, 1997; Banchereau and Steinman, 1998). Several studies have focused on the in vitro generation of DC from haematopoietic progenitors (Caux et al, 1992; Reid et al, 1992; Strunk et al, 1996) and it has been demonstrated that the culture of CD34[+] cells in the presence of GM-CSF and TNF-α, gives rise to CD1a[+]/CD14[–] and CD1a[–]/CD14[+] cell precursors around 6 days of culture, which then differentiate into two subsets including LC (CD1a[+]/E-cadherin[+]) and non-LC (CD1a[+]/E-cadherin[–]), respectively (Caux et al, 1996). The addition of TGF-β1 specifically favours the differentiation of CD1a[+] and CD14[+] precursors into CD1a[+]/E-cadherin[+] LC (Strobl et al, 1996; Caux et al, 1999; Jaksits et al, 1999).

Several studies underline the fact that tumour-soluble factors inhibit the differentiation and/or maturation of generated DC from CD34[+] cells, interfering at different levels of the differentiation pathway, depending on both the histological origin of malignant cells and the cytokine-stimulating cocktail. Indeed, the addition of supernatants from renal cell carcinoma cell lines to GM-CSF/TNF-α stimulated CD34[+] cells blocks the differentiation of both CD14[+]/CD1a[–] and CD14[–]/CD1a[+] precursors by triggering them towards macrophages with a strong phagocytic ability (Menetrier-Caux et al, 1998). Supernatants of breast and colon adenocarcinoma cell lines have been shown to affect the initial steps of DC differentiation from CD34[+] progenitors under GM-CSF/TNF-α/IL-4, resulting in the presence of immature monocytic cells (Gabrilovich et al, 1996). Under the same culture conditions, Shurin et al (2001) also reported that neuroblastoma-derived factors inhibit DC maturation and function. In a similar manner, lung carcinoma conditioned medium was shown to inhibit DC generation and maturation when CD34[+] were cultured in GM-CSF/IL-4 (Katsenelson et al, 2001).

In all epithelial skin tumours and particularly in melanoma, alterations in the number and distribution of epidermal LC have been reported (Stene et al, 1988; Toriyama et al, 1993). In the epidermis overlaying primary melanoma, LC decline in number as melanoma progresses, suggesting that agressive melanoma cells (MC) may affect the generation and/or functional maturation of LC. Here, we provide morphologic, phenotypical and functional evidence that MC differing in their invasive and metastatic properties (Berthier-Vergnes et al, 1993; Zebda et al, 1994a,b; Béchetoille et al, 2000) inhibit the initial steps of LC differentiation from CD34[+] progenitors, but

*Received 20 July 2001*
*Revised 24 September 2001*
*Accepted 25 September 2001*

*Correspondence to:* O Berthier-Vergnes failed to affect the phenotypic and functional maturation of fresh epidermal LC.

### MATERIALS AND METHODS

### Antibodies

The fluorescein isothiocyanate (FITC)-conjugated mouse mAbs used were specific for CD34 (Pharmingen, San José, CA), CD14, CD1a (DAKO, Glostrup, Denmark), CD80, CD83 (Immunotech, Marseille, France), CD86 (Serotec, Oxford, England) and HLA-DR (Caltag Laboratories, Burlingame, CA). The phycoerythrin (PE)-labelled mAb anti-CD1a was purchased from Pharmingen. The unlabelled mAbs included anti-E-cadherin (Takara, Shuzo Co, Shiga, Japan), anti-CD40, anti-CD54, anti-CD58, anti-Class I and anti-Birbeck granules associated Langerin (all from Immunotech). The monoclonal isotype controls were purchased from Sigma Chemical Co (St Louis, MO). Neutralizing specific rabbit polyclonal antibody against VEGF was used at saturating concentrations, as previously described (Guerrin et al, 1995).

### Human melanoma cells

Two well-characterized human melanoma clones IC8 and TIC3 derived from a single parental cell line were used: only T1C3 cells promote lung metastases in animals (Berthier-Vergnes et al, 1993) and penetrate through the dermal–epidermal junction of human skin reconstructs (Bechetoille et al, 2000). These cells were cultured as monolayers in McCoy 5A medium (Gibco, Paisley, Scotland) supplemented with 10% fetal calf serum (FCS) as described previously (Zebda et al, 1994a). Normal human fibroblasts isolated from abdominal skin were cultured as described by Trompezinski et al (2000) and used as controls.

### Generation of human Langerhans cells from cord blood CD34$^+$ progenitors

DC with the phenotypic and functional characteristics of LC were generated as previously described (Caux et al, 1999). Briefly, haematopoietic progenitors were isolated from human umbilical cord blood using a MACS isolation kit (Miltenyi Biotec GmBh, Bergisch Gladbach, Germany), which yielded a purity of more than 90%. CD34$^+$ progenitors were cultured in the presence of 200 U ml$^{-1}$ recombinant human (rh) GM-CSF (specific activity $1.1 \times 10^7$ U mg$^{-1}$; kindly provided by Novartis Pharma, Rueil-Malmaison, France), 25 U ml$^{-1}$ rh TNF-α (specific activity $2 \times 10^7$ U mg$^{-1}$; kindly provided by Dr Ghanem, Institut Bordet, Bruxelles) and 0.5 ng ml$^{-1}$ TGF-β1 (R&D Systems, Abington, UK) in RPMI 1640 (GIBCO, Grand Island, NY) supplemented with 10% fetal bovine serum (FBS, Flow Laboratories, Irvine, UK), $5 \times 10^{-5}$ M 2 mercaptoethanol, penicillin (100 U ml$^{-1}$) and streptomycin (100 μg ml$^{-1}$), referred to as complete medium. Cultured cells were harvested at day 11 and confirmation of LC phenotype was performed using the specific LC markers: E-cadherin, CD1a and Birbeck granules associated Langerin. At least 60% of E-cadherin$^+$ cells gated on high light scatter parameters were obtained in 10 separate experiments.

### Effects of human melanoma cells on the LC generation from CD34$^+$ progenitors

MC ($2 \times 10^5$ cells cm$^{-2}$) and CD34$^+$ progenitors ($4 \times 10^5$ cells cm$^{-2}$) were seeded in complete medium in the lower and upper chamber of 0.4-μm pore size Transwell dishes, respectively (Costar, Cambridge, MA). MC were introduced either at the beginning of culture, at the same time as purified CD34$^+$ cells, or on days 4, 5, 6, 7, 8 of culture. Control experiments were performed in the absence of melanoma cells, or in the presence of normal fibroblasts. In some experiments, CD34$^+$ cells were co-cultured with MC only during the first 7 days of culture, and then cultured alone. In 3 experiments, blocking anti-VEGF antibody was added at saturating concentrations (Guerrin et al, 1995). In all cases, cultured CD34$^+$ cells were collected after 11 days of culture and analysed for phenotype and function. In some cases, CD34$^+$ cells were seeded in the lower chamber, whereas MC were plated in the upper chamber.

### Effect of human melanoma cells on the maturation of fresh epidermal LC

Fresh human epidermal LC were prepared and purified as previously described (Péguct-Navarro et al, 1994). Briefly, epidermal cell suspensions were obtained by trypsinization of human abdominal skin obtained by plastic surgery (0.05% trypsin, Difco Laboratories, Detroit, MI, 1 h at 37˚C). LC were enriched by 2 successive density gradient centrifugations on Lymphoprep (Flobio SA, Courbevoie, France). As assessed by immunofluorescence staining with anti-CD1a mAb, the resulting population routinely contained 75–85% LC. LC ($1 \times 10^6$ cells well$^{-1}$) were placed in the inner transwell chamber and MC ($2 \times 10^5$) in the lower chamber. After a 48 h coincubation, LC were analysed for phenotype and function.

### Immunofluorescence staining procedures and flow cytometry

CD34$^+$ cells cultured for 11 days with or without MC and fresh epidermal LC cultured for 2 days with or without MC were washed in PBS containing 1% FCS, and stained with a panel of FITC-or PE-labelled mouse mAbs or with appropriate isotypic controls. When the mAbs used were unlabelled, cells were subsequently incubated with FITC-conjugated F(ab')$_2$ fragments of goat anti mouse mAb (1:100, Zymed Labs, San Francisco, CA). For double labelling, cells were stained with FITC-conjugated CD14 mAb followed by PE-conjugated anti-CD1a mAb. The cells were analysed by using a FACScan (Becton-Dickinson, Mountain View, CA, USA) and Cell Quest software. The effect of MC was evaluated by the number of positive DC and the MFI, and expressed as the percentage of decrease according to the following formula:

$$\text{\% of decrease} = \frac{\text{MFI of CD34}^+\text{/MC co-culture} - \text{MFI of CD34}^+\text{ cells}}{\text{MFI of CD34}^+\text{ cells alone}} \times 100$$

For each marker, the percentages of decrease of positive cell number and MFI were determined from 4–6 separate experiments. Results were expressed as means ± SD.

### Allogeneic mixed T cell proliferative assays

T cells, obtained from peripheral blood of healthy donors as previously described (Péguet-Navarro et al, 1994), were seeded at $1 \times 10^5$ cells per well into round-bottom 96-well microplates (Nunc, Roskilde, Denmark), and incubated for 5 days in the presence of graded numbers of irradiated (30 Gy) CD34$^+$-derived LC or fresh epidermal LC in 200 μl of RPMI-1640 medium containing 10% human AB serum. T cell proliferation was assessed at day 5, after

*© 2001 Cancer Research Campaign* 

pulsing with [$^3$H]-methyl-thymidine (1 μCi well$^{-1}$; Amersham, Les Ulis, France) for the final 18 h of culture. The incorporated radioactivity was measured by a direct beta-counter (Matrix 96, Packard Instrument Co, Meriden, CT). Results were expressed as the mean cpm ± SD of triplicate cultures.

### Cytokine assays

TGF-β1, IL-10 and VEGF secretion were measured in cell-free culture supernatants of MC, CD34$^+$ cells co-cultured with melanoma cells, and CD34$^+$ cultured alone, using commercially available ELISA kits (R & D Systems Inc, Minneapolis, USA). The quantitative determinations of TGF-β1, IL-10 and VEGF have detection limits of 7 pg ml$^{-1}$, 370 pg ml$^{-1}$ and 5 pg ml$^{-1}$, respectively. All experiments were performed in duplicate and results were expressed as the mean of cytokine per milligram of secreted proteins (ng mg$^{-1}$ ± SD).

### Statistical analysis

The significance of the differences among the percentage of stained cells and their MFI between CD34$^+$ cells cultured with or without MC was determined by the ANOVA for repeated measures and contrasts analysis using JMP™ software (SAS Institute). We also used these procedures for evaluating the differential effect of invasive and non-invasive MC.

### RESULTS

### Altered morphology of CD34$^+$-derived cells upon co-culture with melanoma cells

The typical morphology of cells generated from CD34$^+$ progenitors was strikingly altered when they were co-cultured with human melanoma cells (MC) (Figure 1). CD34$^+$ cells grown in culture medium supplemented with GM-CSF, TNF-α and TGF-β1 formed small non-adherent aggregates surrounded by predominant, scattered adherent cells displaying a dendritic morphology with long projections (Figure 1 A, D). In contrast, CD34$^+$ cells co-cultured with MC from the non-metastatic clone IC8 (Figure 1 B, E) or the metastatic clone T1C3 (Figure 1 C, F) for 7 or 11 days remained small, round-shaped and organized mainly in small clusters. CD34$^+$ progenitors cultured with metastatic or non-metastatic MC, displayed features of undifferentiated, non-adherent cells, distinct from those of macrophages. It should be noted that both melanoma clones did not significantly affect the number and the viability of CD34$^+$-derived cells, as assessed by trypan blue exclusion (data not shown).

### Human melanoma cells disturb the phenotype of CD34$^+$-derived cells

CD34$^+$ progenitors cultured in the presence of GM-CSF, TNF-α and TGF-β1 for 11 days mainly differentiated into typical Langerhans cells (LC), as assessed by high levels of expression of E-cadherin, as previously reported (Caux et al, 1999). They also expressed high level of DC markers such as CD40, CD80, CD54, CD58, CD83, CD86, HLA-DR, HLA-class I. Similar phenotypic profiles were observed when CD34$^+$ cells were cultured in the presence of normal fibroblasts (data not shown). Co-culturing CD34$^+$ cells with MC from the two clones dramatically reduced the percentage of cells positive for CD1a, CD58, CD80, CD83, CD86 and E-cadherin, as well as their respective surface expression level (Figure 2). Whereas the number of cells positive for CD54, CD40, HLA class I and HLA-DR was not significantly affected by melanoma cells, their respective surface expression appeared to be strongly down-regulated (Figure 2).

CD34$^+$ cells co-cultured with MC for 11 days displayed a two- to three-fold increased expression of the monocyte CD14 marker, as assessed by the percentage of positive cells ($P$ = 0.003), as well as their MFI ($P$ = 0.005) compared to CD34$^+$ cells cultured alone (Figure 3). The generation of CD14$^+$ cells was concomitant with a decrease in the percentage of CD1a$^+$ cells ($P$ = 0.001) (Figure 3). No statistical differences were observed when comparing

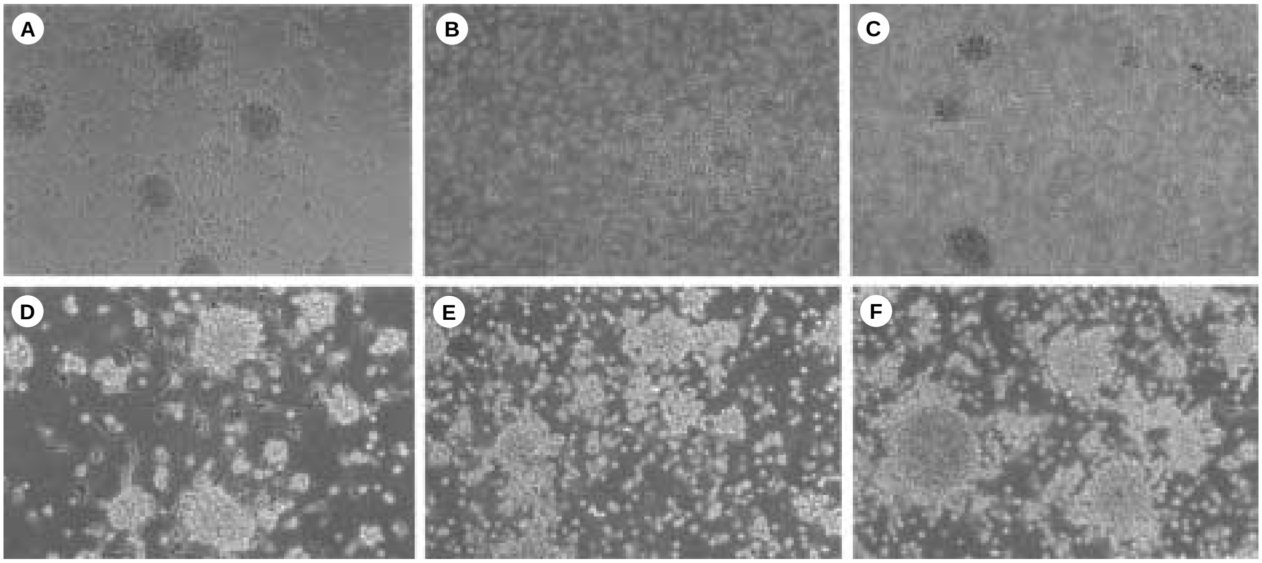

**Figure 1** Altered morphology of CD34$^+$-derived cells upon co-culture with human melanoma cells. CD34$^+$ cells were cultured for 7 days (**A**,**B**,**C**) and 11 days (**D**,**E**,**F**) in medium supplemented with GM-CSF, TNF-α and TGF-β1, either alone (**A**,**D**) or in the presence of human melanoma cells from the non-metastatic clone IC8 (**B**,**E**) or the metastatic clone T1C3 (**C**,**F**), as described in Materials and Methods. These typical phase contrast micrographs obtained from purified CD34$^+$ progenitors from a single donor are representative of 10 different experiments (× 100)

 *© 2001 Cancer Research Campaign*

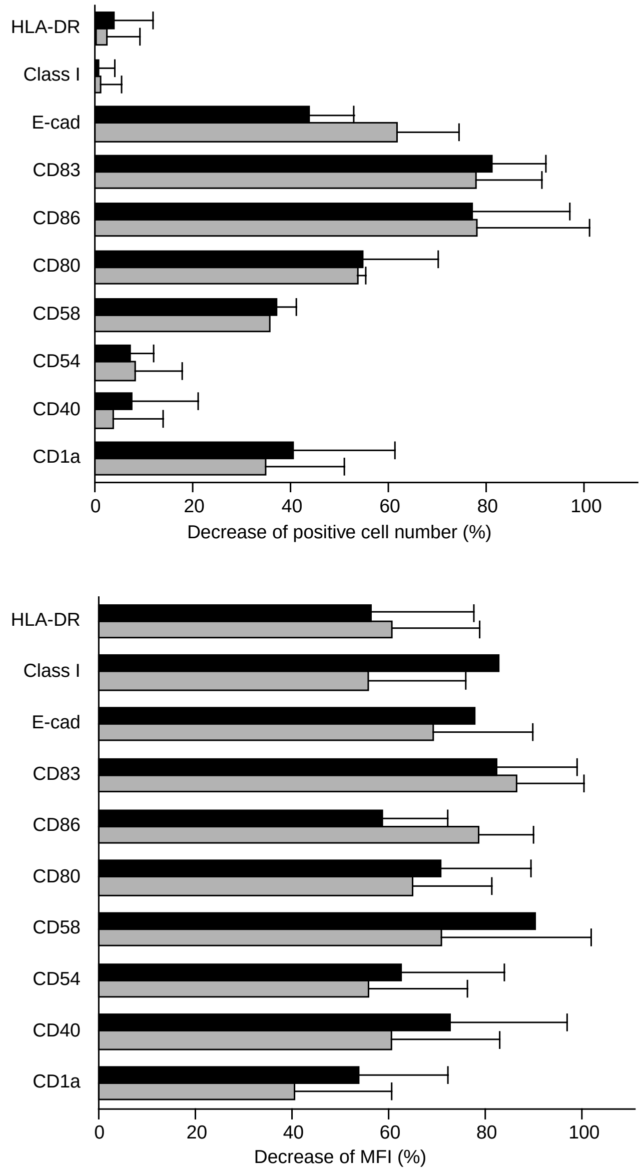

**Figure 2** Melanoma cells modulate the phenotype of cultured CD34⁺ cells. CD34⁺ cells were grown in medium supplemented with GM-CSF, TNF-α and TGF-β1, either alone or in the presence of human melanoma cells from the non-metastatic clone IC8 (■) or with the metastatic clone T1C3 (□). After 11 days of culture, cells were labelled with different mAbs. The effect of melanoma cells was expressed as the percentage of decrease of both the number of positive cells and their respective mean fluorescence intensity (MFI) calculated as indicated in Materials and Methods. Each bar represents the mean ± SD of 5 separate experiments

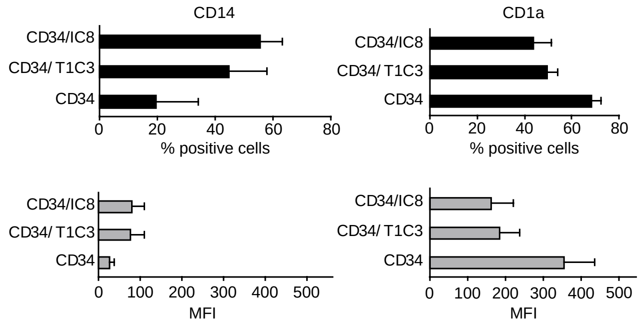

**Figure 3** Melanoma cells lead to an increase in the CD14 population, conversely correlated with a decrease in the CD1a population. CD34⁺ cells were grown in medium supplemented with GM-CSF, TNF-α and TGF-β1, either alone (CD34) or in the presence of human melanoma cells from the IC8 clone (CD34/IC8) or the T1C3 clone (CD34/T1C3). After 11 days of culture, cells were stained with antibodies to CD14 and CD1a. Each bar represents the mean fluorescence intensity (MFI) ± SD or the percentage of positive cells ± SD (%) of 5 separate experiments the effect of melanoma cells with different invasive properties ($P$ = 0.347).

### Human melanoma cells affect the differentiation of CD34⁺ cells only during their early stage of proliferation

We then questioned whether the inhibitory effect of MC on the differentiation of progenitors took place during the expansion or the maturation phase. To this end, CD34⁺ cells were co-cultured with MC either from day 0 to day 7, or from day 8 to day 11 and compared for their phenotype 11 days after initiation of the cultures. A double colour fluorescence labelling for CD1a and CD14 showed that CD34⁺ cells cultured with MC from day 8 to day 11 lacked the CD14 antigen, but mainly expressed CD1a, closely resembling CD34⁺-derived DC grown in the absence of MC (Figure 4A). 5 separate experiments confirm that the expression of CD1a was unmodified (79.4 ± 2.2% with a MFI of 268.9 ± 130 versus 78.9 ± 5% with a MFI of 323.7 ± 158.8). In contrast, the dominance of the CD1a⁺ population over the CD14⁺ population was not found when precursors were cultured with MC either from day 0 to day 11 or day 0 to day 7 (Figure 4A). Cells co-cultured from day 0 to day 7 displayed a decreased expression of CD1a, CD40, CD80, CD83, CD86, E-cadherin, CD54, CD58, HLA-DR and HLA class I at day 11, in a manner similar to that observed in cells co-cultured from day 0 to day 11 (Figure 2). We then evaluated the differential effect of MC on the phenotype of cultured CD34⁺ cells at different time points of their proliferation phase. CD34⁺ cells were grown in GM-CSF, TNF-α and TGF-β1 for 4, 5, 6 and 7 days prior to co-culturing with MC until day 11. As shown in Figure 4B, the earlier the co-culture was started, the more the CD14⁺ population was enhanced. These observations indicate that MC are able to divert the differentiation of cultured CD34⁺ cells towards a dominant CD14⁺ population only when the progenitors are in an early growth phase.

### CD34⁺-derived cells generated in the presence of melanoma cells stimulated T cells less effectively than control LC

To determine whether phenotypic changes of LC precursors co-cultured with MC correlated with altered antigen-presenting function, CD34⁺ cells grown with or without MC were analysed at day 11 for their ability to induce allogeneic T cell proliferation. As shown in Figure 5A, LC generated in vitro from CD34⁺ cells induced significant T cell proliferation. This stimulatory property was not altered when CD34⁺ progenitors were co-cultured with MC from day 8 to day 11 (Figure 5C). In contrast, CD34⁺ progenitors co-cultured with MC, either from day 0 to day 11 (Figure 5A), or even from day 0 to day 7 (Figure 5B), were less potent than control LC in inducing the proliferation of

© 2001 Cancer Research Campaign

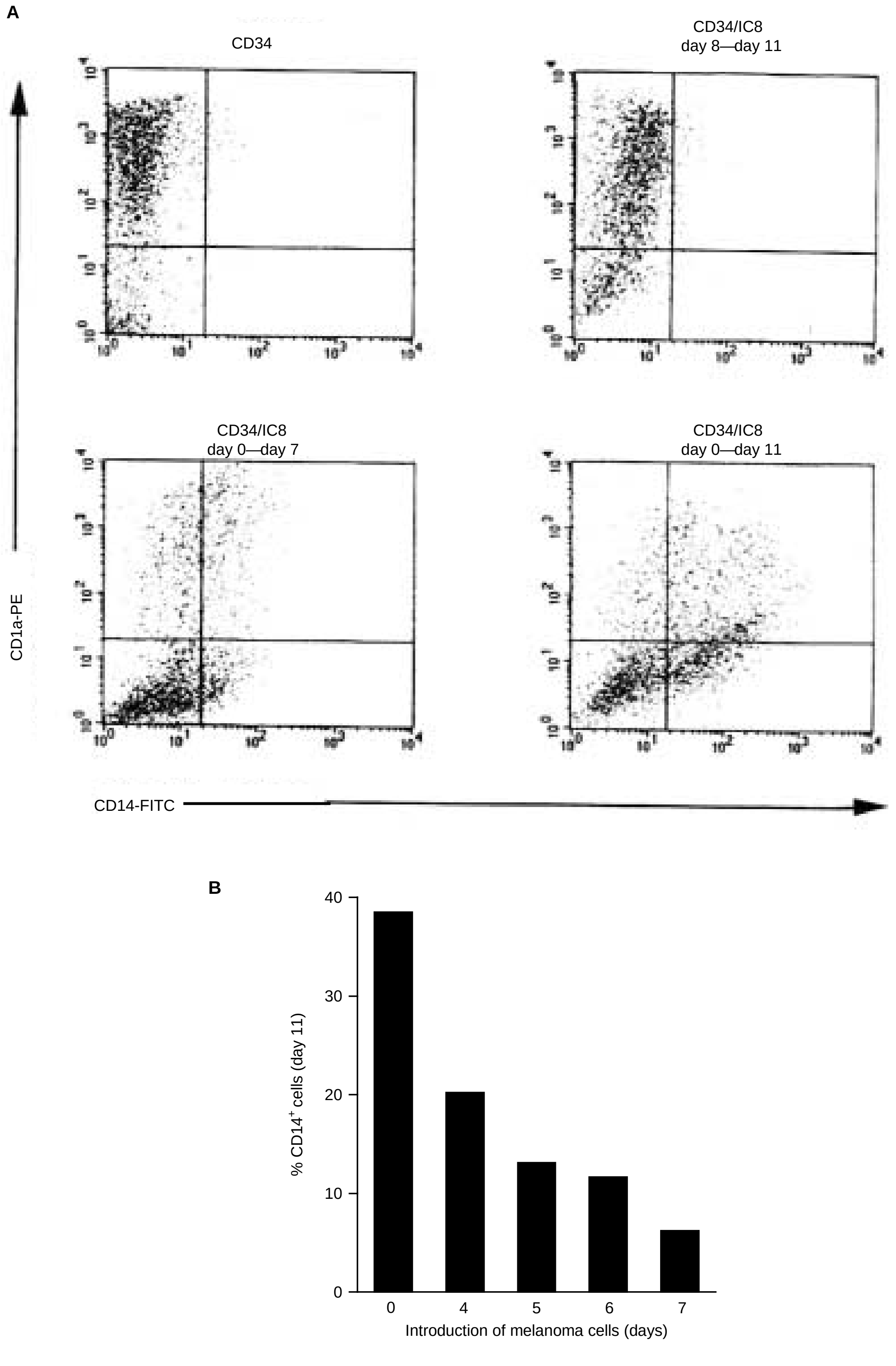

**Figure 4** Melanoma cells direct the differentiation of CD34⁺ cells towards a dominant CD14⁺ population only when progenitors are in an early growth phase. (**A**) CD34⁺ cells were grown in medium supplemented with GM-CSF, TNF-α and TGF-β 1, either alone (CD34) or in the presence of human melanoma cells from the IC8 clone (CD34/IC8). Co-cultures were performed either from day 8 to day 11, day 0 to day 7 or day 0 to day 11, and compared for their expression of CD1a and CD14 11 days after initiation of the cultures. Data are representative of 5 separate experiments. (**B**) CD34⁺ cells were grown in medium supplemented with GM-CSF, TNF-α and TGF-β 1 for 0, 4, 5, 6 and 7 days prior to co-culture with IC8 melanoma cells until day 11. At day 11, the percentage of CD14⁺ cells was evaluated by FACS analysis. In this representative experiment, the percentage of CD14⁺ cells did not exceed 10% when CD34⁺ cells were grown alone for 11 days. Results are representative of 3 separate experiments

 *© 2001 Cancer Research Campaign*

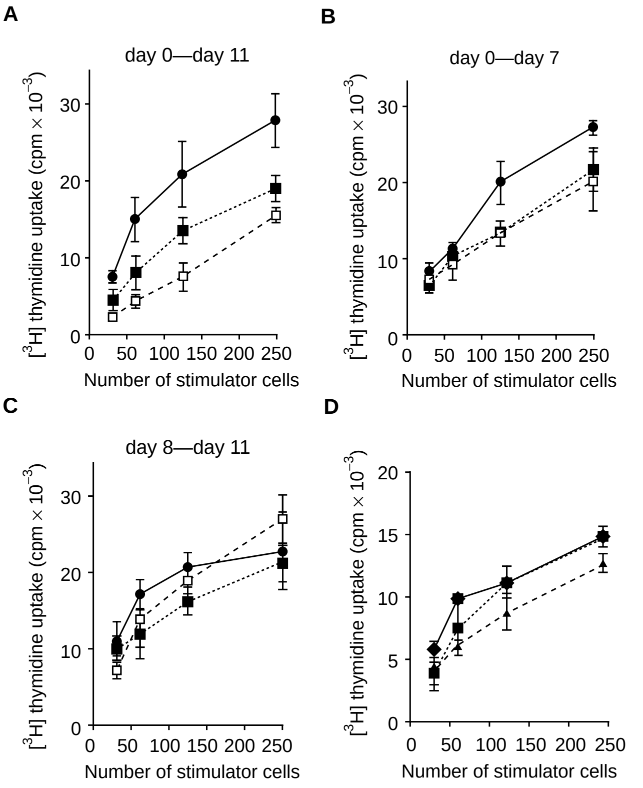

**Figure 5** Melanoma cells decrease the allogeneic T cell proliferation induced by cultured CD34⁺ cells, but do not affect the proliferative T cell response by epidermal LC. CD34⁺ cells were grown in medium supplemented with GM-CSF, TNF-α and TGF-β1, either alone (●) or in the presence of human melanoma cells from the clones IC8 (■) or T1C3 (□). CD34⁺ progenitors were co-cultured with melanoma cells from day 0 to day 11 (**A**), from day 0 to day 7 (**B**) or from day 8 to day 11 (**C**). After 11 days of culture, cells were used as stimulator cells for allogenic T cells ($10^5$ cells well$^{-1}$). Freshly purified LC from the same donor (**D**) were cultured either alone (◆) or in the presence of human melanoma cells from the clones IC8 (■) or T1C3 (▲). After 2 days of culture, LC were used as stimulator cells for allogeneic T cells ($10^5$ cells well$^{-1}$). The proliferation was evaluated by [$^3$H]-thymidine uptake and results are expressed as the mean cpm ± SD of triplicate cultures. The background of T cells alone was 110 ± 30 cpm. Results of each panel are representative of 5 separate experiments allogeneic T cells. Thus, in addition to morphological and phenotypic alterations, CD34⁺ progenitors co-cultured with MC stimulated T cells less effectively than LC generated without MC.

### VEGF, IL-10 and TGF-β1 are not responsible for the inhibition of LC generation

Because VEGF and IL-10 could affect the DC differentiation and/or LC function (Caux et al, 1994; Péguet-Navarro et al, 1994; Gabrilovich et al, 1996), we investigated whether MC could produce these cytokines. When $2 \times 10^5$ MC were plated and grown without CD34⁺ cells, secreted VEGF was found to be 2.9 ± 0.1 ng ml$^{-1}$ 72 h$^{-1}$ for the TIC3 clone and 0.5 ± 0.08 ng ml$^{-1}$ 72 h$^{-1}$ for the IC8 clone. A similar difference in VEGF concentration was found in supernatants collected during the last 72 h of the day 0–day 11 co-cultures of CD34⁺ cells with MC. Under the same culture conditions, IL-10 was not detectable (< 3 pg ml$^{-1}$ 72 h$^{-1}$) in the whole supernatants, even after a 10-fold concentration. In addition, these supernatants were assayed for TGF-β1, known to be critical for LC generation in vitro. The level of TGF-β1 found in supernatants of MC cultured with and without CD34⁺ cells never exceeded that measured in fresh culture medium (0.9 ± 0.03 ng ml$^{-1}$). These data indicate that the melanoma clones under study both secrete VEGF, but not IL-10 or TGF-β1.

To investigate whether VEGF could be responsible for the effect of MC on LC generation, CD34⁺ cells were co-cultured with MC from both clones from day 0 to day 11 in the presence of a neutralizing antibody against VEGF (Guerrin et al, 1995) or a control non-immune rabbit IgG. CD34⁺ cells co-cultured with MC, in the presence or absence of blocking anti-VEGF exhibited a similar pattern of double colour fluorescence staining for CD1a and CD14, as those presented on Figure 4 (day 0–day 11). Thus, the addition of anti-VEGF was not able to abrogate the effect of MC from both clones on the differentiation of cultured CD34⁺ cells. This finding, reproduced in 3 independent experiments, provides evidence that VEGF secreted by the two melanoma clones is not responsible for the inhibition of LC generation.

### Melanoma cells failed to affect the phenotypic and functional maturation of human epidermal LC

Given the close proximity of epidermal LC to the primary site of cutaneous melanoma and their critical role in generating immune response, we then evaluated whether invasive and non-invasive MC may affect the phenotypic and functional maturation of human epidermal LC. To this end, freshly isolated LC from healthy human skin were cultured with or without MC for 2 days. As previously reported (Péguet-Navarro et al, 1995), fresh epidermal LC become fully mature DC and express high levels of HLA-DR, CD40, CD54, CD58, CD80, CD83 and CD86 after a 2 day-period of culture. This phenotypic pattern was unchanged when co-cultured in the presence of MC from both clones or in the presence of normal fibroblasts (data not shown). Moreover, invasive and non-invasive MC were unable to alter the proliferative T cell response induced by allogeneic epidermal LC (Figure 5D). Thus, MC had no effect on the phenotypic and functional maturation of fresh human epidermal LC.

### DISCUSSION

A number of studies have demonstrated that tumour cells can inhibit the differentiation of DC from CD34⁺ progenitors (Gabrilovich et al, 1996; Menetrier-Caux et al, 1998; Katsenelson et al, 2001; Shurin et al, 2001). However, the effect of tumour cells on LC differentiation has never been reported so far. Because of the importance of the LC depletion phenomena in skin melanoma (Stene et al, 1988; Toriyama et al, 1993) and the critical role of LC in generating local immune response, our study was primarily focused on the effect of human melanoma cells on LC. To generate human LC, CD34⁺ cord blood progenitors were cultured in presence of GM-CSF/TNF-α with the addition of TGF-β1, in order to favour the differentiation of these presursors into the particular LC pathway. Under these culture conditions, a population predominantly constituted of epidermal LC was derived on day 11, being characterized by the E-cadherin and CD1a cell surface expression and a high APC function, in agreement with that reported by Caux et al (1999). However, co-culturing of CD34⁺ cells with MC altered their differentiation pathway, leading to the appearance of monocytic CD14⁺ cells. The induction of CD14 antigen expression can be specifically and unequivocally attributed to MC, since it was not observed in the presence of fibroblasts. MC induced a significant increase in the number of CD14⁺ cells which was

*© 2001 Cancer Research Campaign*

correlated with a decrease in the number of CD1a+/E-cadherin+ cells. Cells expressed a reduced level of MHC class II, MHC class I, accessory molecules, and stimulated allogeneic T cells less efficiently than control LC. The induction of apoptotic DC by tumour cell lines has been reported after co-culture of mature CD34+-derived DC (14 days of culture) with tumour cells for a period of 24 – 48 h (Esche et al, 1999; Pirtskhalaishvili et al, 2000). We never observed melanoma-induced apoptosis in CD34+-derived cells under our culture conditions. This could be explained by the fact that TGF-β1 protects cord blood-derived CD34+ cells from apoptosis and increase their survival (Riedl et al, 1997). In addition, CD34+ cells used here were at an earlier stage of differentiation than those used by Esche et al (1999).

MC divert the differentiation of cultured CD34+ cells towards a dominant CD14+ population only during the earliest steps of differentiation (day 0–day 6) which coincides with the proliferative growth period, as previously reported for adenocarcinoma cell lines (Gabrilovich et al, 1996). In addition, the differentiation process leading to the CD14+ phenotype was more efficient when MC were added to CD34+ cultures on day 0, as compared with day 4, 5, 6 or 7. By contrast, CD34+ cells cultured with GM-CSF/TNF-α/TGF-β1 for 7 days before exposure to MC for 4 additional days (day 8 to day 11), has no effect on LC generation and function. Also, MC from both clones had no effect on the phenotype and APC function of freshly isolated epidermal LC, after a 2-day period of culture. Taken together, these data demonstrate that MC specifically affect the earliest differentiation stages of LC precursors, but not the latest stages of in vitro maturation of epidermal LC, from a relatively immature state (fresh LC) to a fully mature functional state. Therefore, it is clear that MC are of critical importance in regulating the number of LC in the skin, by blocking LC generation rather than affecting resident epidermal LC, as it is the case for sarcoma-produced factors towards murine LC (Ishida et al, 1998).

We tested whether in our co-culture system, the tumour factors already identified for their inhibitory effect on DC differentiation, were effective inhibitors on LC differentiation. VEGF produced by breast and colon carcinoma cell lines has been the first identified factor affecting the early stage of CD34+ cell differentiation under GM-CSF/TNF-α/IL-4 (first 4 days of culture), leading to the generation of immature monocytes after 12 days of culture (Gabrilovich et al, 1996). In our co-culture system, VEGF secreted by MC is not responsible for the inhibition of DC differentiation, as demonstrated by blocking studies showing that neutralizing anti-VEGF antibody did not reverse the inhibitory effect. This finding has also been reported for human renal carcinoma cell lines (Ménétrier-Caux et al, 1998). Also, we found that the TIC3 clone produced about 5-fold higher VEGF than the IC8 clone, whereas both clones have quite similar effect on the CD14+ generation. Furthermore, the tumour-released factors including TGF-β1, IL-10, IL-6 and M-CSF, reported to affect DC generation and/or LC function (Caux et al, 1994; Péguet-Navarro et al, 1994; Enk et al, 1997; Menetrier-Caux et al, 1998; Steinbrink et al, 1997) could also be excluded as possible blocking melanoma-derived factors since the respective transcripts of these genes were never detected in the melanoma clones under study, cultured either alone or in the presence of CD34+ cells, as analysed by cDNA array technology (Bertheir-Vergnes et al, manuscript in preparation). In support of this data, neither TGF-β1 nor IL-10 were detected in media from MC co-cultured with CD34+ using specific ELISA. Moreover, only the late differentiation stage (from day 6 to day 12 of culture) has been reported to be affected by IL-6 and M-CSF, which are produced by renal carcinoma cell lines but not by breast carcinoma cell lines (Menetrier-Caux et al, 1998).

Recently, Shurin et al (2001) demonstrated that neuroblastoma-derived gangliosides (GM3 and GD2) inhibit the generation of DC from CD34+ cells. In the situation where GD2 and GM3 are critical factors in blocking LC differentiation, it is unlikely that both melanoma clones would alter LC generation, because of their distinct ganglioside profiles; IC8 melanoma cells mainly express GM3, GM2, GD3 and GD2, whereas TIC3 cells only express low levels of GM3 (Zebda et al, 1994b). Thus, none of the characterized tumour-derived factors able to block the DC differentiation were found to be effective in our co-culture system. A further characterization of the products secreted by MC, responsible for the blockade of LC generation, are currently under investigation.

If our data can be extrapolated to what occurs in vivo, we might speculate that resident epidermal LC, unsensitive to melanoma-derived factors, can play their role of APC by trafficking from the epidermis to regional lymph nodes, inducing a specific immune response. However, it is possible that melanoma-derived factors may play a role at an early LC differentiation stage, and would preferentially prevent the LC generation from their precursors. Thus, CD34+ progenitors leaving the blood circulation and entering the dermis at the tumour site would be unable to generate LC and repopulate the epidermis because of the inhibitory factors secreted by MC at this site. The observations that dermal LC are not found around deeply invasive melanomas (Stene et al, 1988), and that tumour-infiltrating CD1− cells are detected in the dermis deep to melanoma (Toriyama et al, 1993) reinforce this hypothesis. As a consequence, the naturally occurring LC turnover would be severely impaired and would induce a decline in the number of epidermal LC. This hypothesis is supported by immunohistochemical studies demonstrating a decrease in LC number in the epidermis overlying primary melanomas (Stene et al, 1988; Toriyama et al, 1993).

Collectively, our findings clearly demonstrate the differential sensitivity of epidermal LC and their precursors to MC, and suggest that MC may prevent LC generation rather than directly affecting resident epidermal LC in the neighbouring cutaneous microenvironment. These data give new insights on the role of melanoma cells in the local depletion of LC in the epidermis overlying primary melanoma, resulting in an impaired development of anti-tumour immunity leading to tumour progression, rather than a defect in the ability of LC to migrate from the skin to draining lymph nodes.

### ACKNOWLEDGEMENTS

The authors thank Pr Luchini from the Obstetric Division, Saint Joseph Hospital for the supply of umbilical cord blood. The authors are grateful to Drs C Vincent and J Zemmour for critical reading and Mrs Mitchell for revision of the English text. This work was supported by grants from the Ligue Nationale contre le Cancer, Comité Départemental du Rhône.

### REFERENCES

Banchereau J and Steinman RM (1998) Dendritic cells and the control of immunity. *Nature* **392**: 245–252

Béchetoille N, Haftek M, Staquet MJ, Cochran AJ, Schmitt D and Berthier-Vergnes O (2000) Penetration of human metastatic melanoma cells through an authentic dermal–epidermal junction is associated with dissolution of native collagen types IV and VII. *Melanoma Res* **10**: 427–434

*© 2001 Cancer Research Campaign*

Berthier-Vergnes O, Zebda N, Bailly M, Bailly C, Doré JF, Thomas L and Cochran AJ (1993) Expression of peanut agglutinin-binding glycoconjugates in primary melanomas with high risk of metastases. *Lancet* **341**: 1292

Caux C, Dezutter-Dambuyant C, Schmitt D and Banchereau J (1992) GM-CSF and TNF α cooperate in generation of dendritic Langerhans cells. *Nature* **360**: 258–261

Caux C, Massacrier C, Vanbervliet B, Barthelemy C, Liu YJ and Banchereau J (1994) Interleukin 10 inhibits T cell alloreaction induced by human dendritic cells. *Int Immunol* **8**: 1177–1185

Caux C, Vanbervliet B, Massacrier C, Dezutter-Dambuyant C, de Saint-Vis B, Jacquet C, Yoneda K et al (1996) $CD34^+$ hematopoietic progenitors from human cord blood differentiate along two independent dendritic pathways in response to GM-CSF+TNFα. *J Exp Med* **184**: 695–706

Caux C, Massacrier C, Dubois B, Valladeau J, Dezutter-Dambuyant C, Durand I, Schmitt D et al (1999) Respective involvement of TGF-beta and IL-4 in the development of Langerhans cells and non-Langerhans dendritic cells from $CD34^+$ progenitors. *J Leukoc Biol* **66**: 781–791

Enk AH, Jonuleit H, Saloga J and Knop J (1997) Dendritic cells as mediators of tumor-induced tolerance in metastatic melanoma. *Int J Cancer* **73**: 309–316

Esche C, Lokshin A, Shurin GV, Gastman BR, Rabinowich H, Watkins SC, Lotze MT and Shurin MR (1999) Tumor's other immune targets: dendritic cells. *J Leukoc Biol* **66**: 336–344

Gabrilovich DI, Chen HL, Girgis KR, Cunningham HT, Meny GM, Nadaf S, Kavanaugh D and Carbone DP (1996) Production of vascular endothelial growth factor by human tumors inhibits the functional maturation of dendritic cells. *Nat Med* **2**: 1096–1103

Guerrin M, Moukadiri H, Chollet P, Moro F, Dutt K, Malecaze F and Plouët J (1995) Vasculotropin/vascular endothelial growth factor is an autocrine growth factor for human retinal pigment epithelial cells. *J Cell Physiol* **164**: 385–394

Hart DNJ (1997) Dendritic cells: unique leukocyte populations which control the primary immune response. *Blood* **90**: 3245–3287

Ishida T, Oyama T, Carbone DP and Gabrilovich DL (1998) Defective function of Langerhans cells in tumor-bearing animals is the result of defective maturation from hemopoietic progenitors. *J Immunol* **161**: 4842–4851

Jaksits S, Kriehuber E, Charbonnier AS, Rappersberger K, Stingl G and Maurer D (1999) $CD34^+$ cell-derived $CD14^+$ precursor cells develop into Langerhans cells in a TGF-beta-dependent manner. *J Immunol* **163**: 4869–4877

Katsenelson NS, Shurin GV, Bykovskaia SN, Shogan J and Shurin MR (2001) Human small cell lung carcinoma and carcinoid tumor regulate dendritic cell maturation and function. *Mod Pathol* **14**: 40–45

Menetrier-Caux C, Montmain G, Dieu MC, Bain C, Favrot MC, Caux C and Blay JY (1998) Inhibition of the differentiation of dendritic cells from $CD34^+$ progenitors by tumor cells: role of Interleukin-6 and macrophage colony-stimulating factor. *Blood* **92**: 4778–4791

Péguet-Navarro J, Moulon C, Caux C, Dalbiez-Gauthier C, Banchereau J and Schmitt D (1994) Interleukin-10 inhibits the primary allogeneic T cell response to human epidermal Langerhans cells. *Eur J Immunol* **24**: 884–891

Péguet-Navarro J, Dalbiez-Gauthier C, Rattis FM, Van Kooten C, Banchereau J and Schmitt D (1995) Functional expression of CD40 antigen on human epidermal Langerhans cells. *J Immunol* **155**: 4241–4247

Pirtskhalaishvili G, Shurin GV, Esche C, Cai Q, Salup RR, Bykovskaia SN, Lotze MT and Shurin MR (2000) Cytokine-mediated protection of human dendritic cells from prostate cancer-induced apoptosis is regulated by the Bcl-2 family of proteins. *Br J Cancer* **83**: 506–513

Reid CD, Stackpoole A, Meager A and Tikerpae J (1992) Interactions of tumor necrosis factor with granulocyte-macrophage colony-stimulating factor and other cytokines in the regulation of dendritic cell growth in vitro from early bipotent $CD34^+$ progenitors in human bone marrow. *J Immunol* **149**: 2681–2688

Riedl E, Strobl H, Majdic O and Knapp W (1997) TGF-beta 1 promotes in vitro generation of dendritic cells by protecting progenitor cells from apoptosis. *J Immunol* **158**: 1591–1597

Shurin GV, Shurin MR, Bykovskaia S, Shogan J, Lotze MT and Barksdale EM Jr. (2001) Neuroblastoma-derived gangliosides inhibit dendritic cell generation and function. *Cancer Res* **61**: 363–369

Steinbrink K, Wolfl M, Jonuleit H, Knop J and Enk AH (1997) Induction of tolerance by IL-10-treated dendritic cells. *J Immunol* **159**: 4772–4780

Stene MA, Babajanians M, Bhuta S and Cochran AJ (1988) Quantitative alterations in cutaneous Langerhans cells during the evolution of malignant melanoma of the skin. *J Invest Dermatol* **91**: 125–128

Strobl H, Riedl E, Scheinecker C, Bello-Fernandez C, Pickl WF, Rappersberger K, Madjic O and Knapp W (1996) TGF-β1 promotes in vitro development of dendritic cells from $CD34^+$ hemopoietic progenitors. *J Immunol* **157**: 1499–1507

Strunk D, Rappersberger K, Egger C, Strobl H, Kromer E, Elbe A, Maurer D and Stingl G (1996) Generation of human dendritic cells/Langerhans cells from circulating $CD34^+$ hematopoietic progenitor cells. *Blood* **87**: 1292–1302

Toriyama K, Wen DR, Paul E and Cochran AJ (1993) Variations in the distribution, frequency, and phenotype of Langerhans cells during the evolution of malignant melanoma of the skin. *J Invest Dermatol* **100**: 269s–273s

Trompezinski S, Pernet I, Mayoux C, Schmitt D and Viac J (2000) Transforming growth factor-betal and ultraviolet A1 radiation increase production of vascular endothelial growth factor but not endothelin-1 in human dermal fibroblasts. *Br J Dermatol* **143**: 539–545

Zebda N, Bailly M, Brown S, Doré JF and Berthier-Vergnes O (1994a) Expression of PNA-binding sites on specific glycoproteins by human melanoma cells is associated with a high metastatic potential. *J Cell Biochem* **54**: 161–173

Zebda N, Pedron S, Rebbaa A, Portoukalian J and Berthier-Vergnes O (1994b) Deficiency of ganglioside biosynthesis in metastatic human melanoma cells: relevance of CMP-NeuAc: LacCer α2,3 sialyltransférase (GM3 synthase). *FEBS Letters* **362**: 161–164

© 2001 Cancer Research Campaign
